# Personality Traits as Mediators of the Relationship Between Affective Distress and Self-Esteem Among Incarcerated Youth: A Parallel Mediation Model

**DOI:** 10.3390/bs16101891

**Published:** 2026-10-13

**Authors:** Cornelia Rada, Suzana Turcu, Flavia-Elena Ciurbea, Adela-Mihaela Țăranu

**Affiliations:** 1 Francisc I. Rainer Institute of Anthropology, Romanian Academy, 050474 Bucharest, Romania; corneliarada@yahoo.com; 2 Penitentiary Inspection Directorate, National Administration of Penitentiaries, 023761 Bucharest, Romania; ciurbeaflavia@gmail.com; 3 Department of Teacher Training, Technical University of Civil Engineering of Bucharest, 020396 Bucharest, Romania; adela.taranu@ise.ro; 4 Institute of Educational Sciences, 010102 Bucharest, Romania

**Keywords:** incarcerated youth, affective distress, self-esteem, personality traits, Big Five personality model, mediation analysis

## Abstract

This study examined how affective distress, self-esteem, and personality traits are related among detained young adults, and how these factors connect to rehabilitation-relevant risk factors. The sample included 455 participants (18–25 years, M = 21.31, SD = 2.32; 97.8% male), recruited from educational, detention and prison centers. CP5F, RSES, and PDA were used, and analyses included Mann–Whitney tests, linear regression, and mediation models. The results showed that placement history predicted higher levels of Depression and Total Distress, as well as lower self-esteem, while access to intimate visits was associated with lower levels of Sadness and Total Distress. The diagnosis of chronic/psychiatric illness, suicide attempts, and trauma in the family of origin were associated with higher levels of distress, while diagnosis, suicide attempts, and a history of recidivism were also associated with lower self-esteem. In the regression model, Depression was negatively associated with self-esteem, whereas Concern showed a positive association (*R*^2^ = 0.18). In the parallel mediation model, the Dysfunctional Score was negatively associated with self-esteem both directly and indirectly, through Extraversion, Conscientiousness and Emotional Stability; Autonomy showed no mediating effect and the indirect effect of Agreeableness, significant in the individual model, became non-significant when tested alongside the other mediators. The total indirect effect and the direct effect were both significant in the parallel mediation model. The conclusions highlight the central role of affective distress in lowering self-esteem and support the need for early psychological screening and detention interventions aimed at reducing distress and strengthening emotional stability.

## 1. Introduction

Stress is an inevitable component of psychological life and can have both adaptive effects and negative consequences on the functioning of the individual. While eustress can support development and adaptation, prolonged exposure to intense stressors can generate affective distress, associated with deterioration in physical and mental health (Ghasemi et al., 2024).

In the context of judicial proceedings and deprivation of liberty, distress can be amplified, being influenced by both institutional pressures and individual emotional experiences. Research has shown that health status can deteriorate with prolonged exposure to court proceedings (Clemente & Padilla-Racero, 2020), and first-time incarcerated individuals are more vulnerable to developing depressive symptoms (D’Orta et al., 2022). The deprivation of liberty environment is described in the literature as unpredictable and tense, with newcomers facing significant social and emotional challenges (Enga, 2024), to which are added guilt, shame and self-blame (Lamb, 2003). In this context of emotional vulnerability associated with detention, the role of self-esteem has been frequently investigated in the literature.

In parallel, the scientific literature on the relationship between self-esteem and delinquency highlights uneven results. Some theoretical perspectives argue that the low level of self-evaluation favors delinquent behaviors, while other approaches point to opposite directions of the relationship (Frank, 2004; Oser, 2006; Ostrowsky, 2010). Meta-analysis by Mier and Ladny (2017) suggested that self-esteem explains criminal behaviors only to a limited extent, but more recent research has highlighted the adaptive role of stable self-esteem associated with lower levels of antisocial and risky behaviors (Orth & Robins, 2022; Martínez-Casanova et al., 2024).

An important aspect is the conceptual distinction between healthy self-esteem and narcissism. Healthy (or optimal) self-esteem, as referred to in this study, denotes a relatively stable and non-contingent sense of self-worth, as opposed to fragile, defensive or contingent forms of high self-esteem, which are more closely associated with narcissistic self-enhancement (Kernis, 2003). While stable self-esteem correlates with psychological adaptation and reduced externalizing behaviors, narcissism is associated with grandiosity, insensitivity, and destructive attitudes (Hyatt et al., 2018). While both imply a positive self-image, narcissism involves exaggerated and rigid self-evaluation (Brummelman et al., 2016; Southard et al., 2018; Burger & Mortimer, 2024). In general, individuals with high and stable self-esteem exhibit a more effective adaptation to difficulties and more satisfying social relationships (Jiang et al., 2024), but the literature has also highlighted the existence of a “dark side” of self-esteem characterized by exaggerated self-worth associated with violent behaviors (Baumeister et al., 1996; Walker & Bright, 2009; Rada & Forțu, 2026).

Building on this literature, we argue that in the penitentiary environment, the dynamics between affective distress, self-esteem and personality traits warrant particular attention, given that psychological adaptation during detention is likely shaped jointly by individual dispositions and contextual constraints specific to incarceration. The relationships between these variables are complex and cannot be explained by simple associations, as criminal behavior involves both psychological dimensions and social and situational determinants.

The need for the present study derives from the need for a complex approach that simultaneously examines affective distress, self-esteem, personality traits and vulnerability factors specific to the detention environment. The study contributes to the literature by developing a trauma factor score, testing a mediation model that investigates the effect of affective distress on self-esteem through major personality traits within the Big Five model, and collecting data from most of the data directly from detention facilities.

In this context, the study aimed to investigate the relationship between affective distress, self-esteem and major personality traits in young adults deprived of liberty, as well as to examine the association of these dimensions with psychosocial vulnerability factors relevant to the rehabilitation process.

The selection of personality traits as candidate mediators is grounded in two converging lines of evidence. First, a large-scale meta-analysis has shown that the Big Five dimensions—particularly low Emotional Stability (Neuroticism) and low Conscientiousness—are robust correlates of depressive, anxiety and substance-use symptomatology (Kotov et al., 2010), supporting their relevance as pathways through which affective distress may translate into broader psychological dysfunction. Second, in one of the largest studies examining the personality basis of self-worth (*N* = 326,641), Robins et al. (2001) found that the Big Five jointly accounted for approximately 34% of the variance in self-esteem, with Emotional Stability, Extraversion, and Conscientiousness emerging as the strongest and most consistent correlates, while Agreeableness showed a comparatively weaker and less stable association. This pattern anticipates, and is later closely mirrored by, the present study’s finding that Extraversion, Conscientiousness, and Emotional Stability retained significant mediating effects in the parallel model, whereas the effect of Agreeableness became non-significant once shared variance with the other traits was accounted for.

In line with the formulated objectives, the study aimed to test several hypotheses regarding the relationships between affective distress, self-esteem and major personality traits in the context of detention. Thus, it was assumed that contextual factors associated with psychosocial vulnerability, such as placement history, diagnosis of chronic or psychiatric conditions, traumatic experiences, suicide attempts, relapse and access to intimate visits, are differentially associated with the level of affective distress and self-esteem (H1). It has also been anticipated that the dimensions of affective distress present distinct relationships with self-esteem, reflecting different contributions of functional and dysfunctional negative emotions on self-perception (H2). In addition, the role of major personality traits in the dynamics between affective distress, positive emotionality and self-esteem was investigated, these being considered both direct predictors of self-worth and mediating mechanisms of the relationship between dysfunctional affective distress and self-esteem (H3). Specifically, given the documented associations between the Big Five dimensions and both affective symptomatology and self-esteem (Kotov et al., 2010; Robins et al., 2001), it was hypothesized that Extraversion, Conscientiousness, Agreeableness and Emotional Stability would each show direct positive associations with self-esteem, and that Emotional Stability, Extraversion and Conscientiousness would mediate the negative relationship between dysfunctional affective distress and self-esteem.

## 2. Materials and Methods

### 2.1. Study Design

The study used a quantitative methodological approach to investigate the relationships between affective distress, self-esteem, and major personality traits defined by the Big Five model among young adults in detention. As part of the broader doctoral research programme, 32 semi-structured interviews were also conducted with participants; these qualitative data are analyzed separately and are not part of the present quantitative analysis. The data were collected between 2021 and 2024 by administering assessment tools in pencil–paper format.

Access to the penitentiary environment involved obtaining the agreement of the National Administration of Penitentiaries and collaboration with the management of the units involved the research being carried out according to the specific program of each detention sector. Participants were individually invited to complete the battery of questionnaires after participating in the training on the research procedure. The research was approved by the Ethics Committee of the Institute of Philosophy and Psychology “Constantin Rădulescu-Motru”, Romanian Academy, Bucharest (Certificate no. 80/19.03.2021) and complied with the principles of the Declaration of Helsinki. Data collection began after obtaining preliminary approval from the National Administration of Penitentiaries (no. 7786/29.04.2021).

The participants signed the informed consent and were informed about the purpose of the study, data privacy and compliance with European and national data protection standards, as well as the possibility of withdrawal from the research at any time without consequences.

### 2.2. Participants and Data Collection

The convenience sample consisted of 455 young adults deprived of liberty, recruited from the entire national network of institutions responsible for executing custodial educational measures for minors and young people (the two educational centers, Buziaș and Târgu Ocna and two detention centers, Brăila-Tichilești and Craiova), as well as from five penitentiaries across Romania (National Administration of Penitentiaries, Subordinate Units, 2021).

The initial sampling frame targeted young people up to 21 years of age held in educational and detention centres. This was subsequently broadened to include eligible individuals held in penitentiaries, since only a subset of convicted or remanded youth are held in educational/detention centres, the remainder being held in penitentiaries. The upper age limit was extended to 25 years to account for individuals who committed offences as minors but were older at the time of data collection, consistent with national legislation permitting the continuation of educational measures within a penitentiary setting for individuals over 18 who negatively influence other residents (Parlamentul României, 2013). This extension was further supported by findings indicating that most recidivists in the Romanian prison population committed their first offence between the ages of 14 and 25 (Scutaru et al., 2019; Ministerul Justiţiei, 2024).

Participants’ ages ranged from 18 to 25 years (M = 21.31, SD = 2.32) and the majority were male (97.8%). Only one participant did not provide their date of birth.

Following institutional approval, participation was announced within each facility as a voluntary opportunity, and all young people who expressed interest were enrolled after providing informed consent. No additional inclusion criteria beyond willingness to participate and capacity to complete the assessment battery (with interview-format administration offered to non-literate participants) were applied. Of the 487 questionnaires collected, 32 were excluded prior to analysis due to data-quality concerns, including extensive missing responses, patterned or careless responding (e.g., uniform selection of a single response option across an entire instrument), and implausible demographic values (e.g., inconsistent or impossible reported age), yielding a final analytic sample of *N* = 455. The exact number of individuals who were informed about the study but declined participation was not systematically recorded across all facilities and constitutes a limitation of the present study, precluding calculation of a participation/response rate. Data were collected using different administration modalities across facilities: by the doctoral researcher (in person at most sites, and remotely at one educational centre), and, at three penitentiary units, by institutional staff (psychologists, educators) following the researcher’s protocol. This variation in administrator status could have introduced differential social desirability effects, particularly relevant given that the CP5F includes a dedicated Social Desirability scale; this potential source of variability was not formally tested and is acknowledged as a limitation.

### 2.3. Variables and Measurements

The data were collected through a standardized set of psychometric tools: the 5-Factor Personality Questionnaire (CP5F) (Albu & Porumb, 2009), a tool built on the Big Five paradigm and used to assess the major dimensions of personality, the Rosenberg Self-Esteem Scale (RSES) (Robu, 2013) and the Affective Distress Profile (PDA) (Opriș & Macavei, 2007a). In addition, a number of contextual variables relevant to the detention environment were analyzed, assessed through self-report items: the existence of a diagnosis of chronic or psychiatric illness, the exercise of the right to intimate visitation, placement history, criminal recidivism, traumatic experiences (especially negative events in the family of origin) and previous suicide attempts.

Childhood adversity within the family of origin was assessed using an 11-item ad hoc checklist of dichotomous (yes/no) items, modeled on the Adverse Childhood Experiences (ACE) framework (Felitti et al., 1998; Hughes et al., 2017). Items 7, 8, and 10 were reverse-scored, as they described protective rather than adverse conditions (e.g., parental interest in schooling). Responses were summed and min–max-normalized to a continuous score ranging from 0 to 1 (TRAUMA). For group-comparison analyses, participants were dichotomized at a threshold of zero on the normalized score, with a score of zero (no adverse experiences endorsed) classified as the no-trauma group. Internal consistency of the 11 items, assessed using the Kuder–Richardson Formula 20 (KR-20), was modest (KR-20 = 0.622), consistent with the heterogeneous, checklist-based nature of ACE-type indices, which assess breadth of exposure to distinct types of adversity rather than intensity of a single underlying construct.

Personality traits were assessed with the 5-Factor Personality Questionnaire (CP5F; Albu & Porumb, 2009), an instrument based on the Big Five paradigm, the scales evaluated being: Autonomy, Extraversion, Agreeableness, Conscientiousness and Emotional Stability. The questionnaire also includes a control scale for Social Desirability. The tool comprises 130 items rated on a five-step Likert scale. Negatively worded items were recoded before the scores were calculated, and the raw scores were aggregated for each personality dimension. The scores were subsequently transformed into T-scores based on the Romanian benchmarks, allowing the interpretation of the personality profile in relation to the norms of the reference population, and were used in regression and mediation analyses to examine the relationships between major personality traits, affective distress and self-esteem.

Self-esteem was assessed using the Rosenberg Scale (RSES), one of the most widely used tools for measuring this construct (Miller, 2020). The scale contains ten items formulated both positively and negatively, rated on a four-point Likert scale, with the total score ranging from 10 to 40. Internal consistency was assessed by the Cronbach coefficient α (α = 0.664; 95% CI [0.607, 0.704]), indicating a modest value; deleting item 3 would result in an acceptable value (α = 0.718). Additional coefficients indicated values ω = 0.640 (95% CI [0.463, 0.715]) and Guttman λ2 = 0.702 (95% CI [0.662, 0.739]).

**Figure 1:**
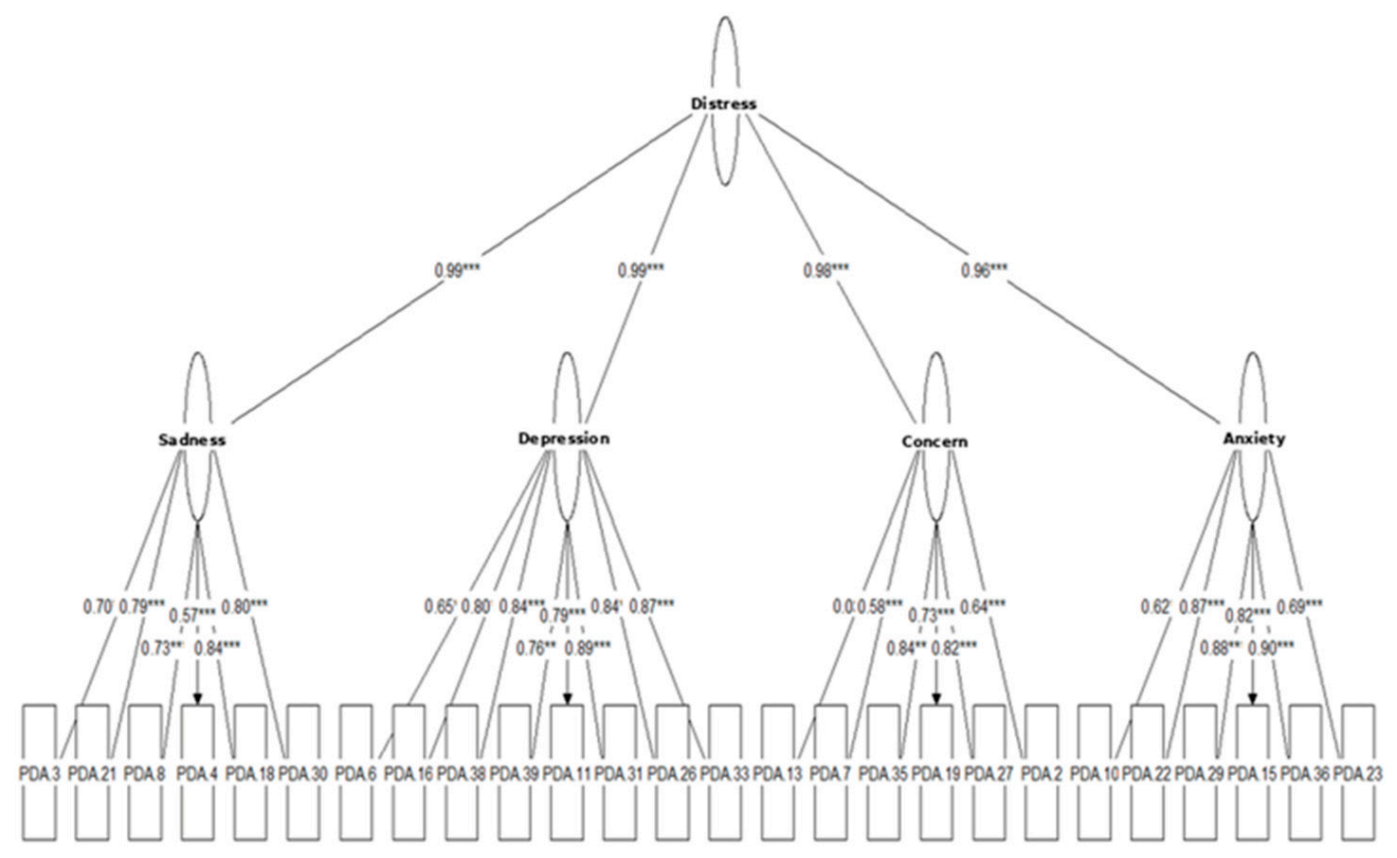
Statistical model for Total Distress; hierarchical structure. Note: ** *p* < 0.01; *** *p* < 0.001.

The Affective Distress Profile (PDA) was adapted in Romania in 2005 (Opriș & Macavei, 2005) and allows scores for Total Distress, Positive Emotions, Concern, Anxiety, Sadness and Depression. The grading of the items was carried out according to the methodology described by Opriș and Macavei (2007b), directly for negative emotions (from 1—Not at all to 5—Very much) and indirectly for Positive Emotions (from 1—Very much to 5—Not at all). The aggregate scores included the Functional Score (functional negative emotions—12 items) and Dysfunctional Score (dysfunctional negative emotions—14 items) subscales, as well as the Total Distress score (26 items) (Figure 1). The internal consistency of the scales was analyzed, obtaining Cronbach values α between 0.738 and 0.953. The factor structure was tested by confirmatory factor analysis (CFA), the hierarchical model of Total Distress presenting satisfactory match indicators (CFI = 0.996; TLI = 0.995; RMSEA = 0.054; pRMSEA = 0.143). The Positive Emotions construct was confirmed by a separate CFA model (CFI = 0.988; TLI = 0.980).

### 2.4. Analytical Strategy

Statistical analyses were performed using non-parametric tests and regression models, depending on the characteristics of the data distributions. The differences between the groups were examined by the Mann–Whitney U test, used to compare the scores obtained on the PDA scales, in relation to the placement history, the exercise of the right to intimate visitation, the existence of suicide attempts in the past and the experience of trauma within the family of origin, as well as the scores obtained on the Rosenberg scale (RSES), in relation to the placement history, the state of relapse, the existence of a diagnosis of chronic or mental illness and the existence of previous suicide attempts. The choice between parametric and non-parametric procedures was made on a per-analysis basis: non-parametric tests were used for between-group comparisons where preliminary inspection indicated departures from normality and/or unequal group sizes, whereas regression and mediation models were retained because these methods require normally distributed residuals rather than normally distributed raw scores, a condition that was verified separately for each model (Section 3.3 and Section 3.7).

The mediating role of personality traits assessed by CP5F was tested by mediation models, analyzing the relationship between Dysfunctional Score (PDA) and Self-Esteem (RSES), with centered scores, WLS estimator and Bonferroni adjustment (separate models and parallel mediation) (Kenny, 2025). Additional regression analyses were also performed to examine the relationship between Positive Emotions (PDA) and personality dimensions (CP5F), as well as the relationship between self-esteem (RSES) and the traits investigated by CP5F.

### 2.5. Sample Size and Power Analysis

The required sample size was informed a priori by non-specific G*Power (Faul et al., 2007) and Soper (2025) calculations conducted at the planning stage of the broader doctoral research programme (G*Power 3). For the confirmatory hierarchical factor model of Total Distress (Figure 1), post-hoc power analysis conducted using the semPower package in R (*N* = 332, df = 167, α = 0.05), testing the model’s ability to distinguish close fit (RMSEA ≤ 0.05) from not-close fit (RMSEA ≥ 0.08), indicated a power exceeding 0.99. For the parallel mediation model, a priori power analysis for a model with four mediators indicated power exceeding 95% for samples above *N* = 295; a sensitivity analysis based on the *N* = 318 complete cases retained in this model indicated that indirect effects of the magnitude reported could be detected with power exceeding 95%. While the final sample of 455 participants exceeded the thresholds required for the univariate tests reported in this study, listwise deletion due to missing data reduced the analytic sample for the confirmatory factor and mediation models (*N* = 332 and *N* = 318, respectively); as shown above, statistical power for these specific models remained high despite this reduction.

## 3. Results

### 3.1. Descriptive Statistics

In the analyzed sample, 399 people fully answered the RSES. The minimum score was 12, the maximum score 40 with a statistical average of 30.4 and with a standard deviation of 4.7.

Descriptive statistics on each PDA scale are presented in Table 1.

For CP5F, the acceptable Cronbach Alpha value greater than 0.7 (Bland & Altman, 1997) was observed only on the Social Desirability, Conscientiousness, Agreeableness, and Emotional Stability scales. Consistency was poor on the Extraversion (0.6) and Autonomy (0.6) scales.

The statistical results for the CP5F scales indicated different mean values between the personality dimensions analyzed. The highest average scores were observed for Conscientiousness (M = 90.144), followed by Agreeableness (M = 83.761), Autonomy (M = 74.534), Emotional Stability (M = 73.519), and Extraversion (M = 70.078), while Social Desirability showed an average of 56.79. The variability in the scores was moderate, with standard deviations ranging from 10.049 (Extraversion) to 15.105 (Conscientiousness). The score ranges indicated a wide dispersion within the sample, with minimum values ranging from 1.00 (Emotional Stability) to 30.00 (Conscientiousness), and maximum values between 75.00 (Social Desirability) and 173.00 (Autonomy), suggesting a relatively high heterogeneity of personality profiles in the analyzed group.

### 3.2. Group Differences (Mann–Whitney)

Considering the non-fulfillment of the normality hypotheses, non-parametric Mann–Whitney U tests were used to test the differences between the groups according to the contextual variables investigated.

#### 3.2.1. Mann–Whitney Tests Applied to PDA Scales

Participants with a history of placement had significantly higher scores in Depression (Z = −2.068, *p* = 0.039) and Total Distress (Z = −2.090, *p* = 0.037), while the difference for Sadness was marginally significant (Z = −1.934, *p* = 0.053).

Depending on the experience of intimate visits, young people who benefited from such visits recorded significantly lower scores in Sadness (Z = −2.498, *p* = 0.012) and Total Distress (Z = −2.228, *p* = 0.026).

Participants diagnosed with chronic or psychiatric illness scored significantly higher on all dimensions of the negative emotions assessed (Sadness, Depression, Concern, Anxiety, and Total Distress; all *p* < 0.001).

No significant differences were observed between the scores of drug users and non-users.

Participants with a history of suicide attempts also had significantly higher levels of all negative emotions analyzed, including Sadness (Z = −4.028, *p* < 0.001), Depression (Z = −4.399, *p* < 0.001), Concern (Z = −4.455, *p* < 0.001), Anxiety (Z = −4.680, *p* < 0.001), and Total Distress (Z = −4.057, *p* < 0.001).

In terms of trauma history, participants who reported traumatic experiences scored significantly higher on Sadness (Z = −2.576, *p* = 0.010), Depression (Z = −3.352, *p* < 0.001), and Total Distress (Z = −2.591, *p* = 0.010).

#### 3.2.2. Mann–Whitney Test Results for the Rosenberg Self-Esteem Scale (RSES)

The analysis of the RSES scores revealed significant differences depending on the clinical and judicial variables investigated. Young people with no placement history had higher self-esteem scores (Average rank = 201.02) compared to those who grew up in foster care (Average rank = 159.27), with the difference being statistically significant (Z = −2.380, *p* = 0.017).

Also, first-conviction participants had significantly higher RSES scores (Average rank = 200.23) than repeat offenders (Average rank = 163.63; Z = −2.875, *p* = 0.004).

Depending on medical status, participants without a diagnosis of chronic or psychiatric illness achieved significantly higher self-esteem scores (Average rank = 205.47) compared to those diagnosed (Average rank = 145.77; Z = −3.228, *p* = 0.001).

Similarly, the absence of a suicidal history was associated with higher self-esteem scores (Average rank = 204.95) compared to participants with previous suicide attempts (Average rank = 161.90; Z = −2.742, *p* < 0.006).

### 3.3. Relationship Between RSES and PDA (Linear Regression)

To examine the relationship between RSES scores and affective distress (PDA) dimensions, a linear regression analysis was performed. The results indicated that the RSES scores were significantly predicted by the Concern and Depression dimensions, *F*(2, 324) = 37.6, *p* < 0.001, with the model explaining 18% of the self-esteem variability (*R*^2^ = 0.18).

The significant coefficients of the regression model, together with the null model intercept (M_0_) and the parameters of the predictor model (M_1_), are presented in Table 2.

The coefficient of the predictor Concern was significantly positive (0.197). The increase in the Concern score caused the RSES score to increase. The Depression score coefficient was significantly negative, indicating that if the depression score increases by one unit, the self-esteem score decreases by 0.326 units. The other predictors (Sadness, Anxiety and Positive Emotions) were not significant. VIF values below the reference value (VIF = 5) did not indicate collinearity problems.

The normality hypothesis was verified by inspection of the Q-Q graphs and the Kolmogorov–Smirnov (*p* = 0.51) and Shapiro–Wilk (*p* = 0.08) numerical tests. The Breusch–Pagan (*p* = 0.44), Goldfeld–Quandt (*p* = 0.58) and Harrison–McCabe (*p* = 0.585) tests indicated that the homoscedasticity hypothesis was also satisfied. The Durbin–Watson test for autocorrelations (DW = 1.87, *p* = 0.264) was insignificant.

### 3.4. Mediation of the Dysfunctional Score (PDA) → RSES Through CP5F

In this mediation analysis, we examined the extent to which personality traits (CP5F) mediate the relationship between Dysfunctional (negative emotions) score (PDA) and self-esteem (RSES). Five mediation models were tested, corresponding to each personality trait considered mediator. Centered scores, the WLS estimator and the Bonferroni correction were used in the analysis to control the type I error.

**Figure 2:**
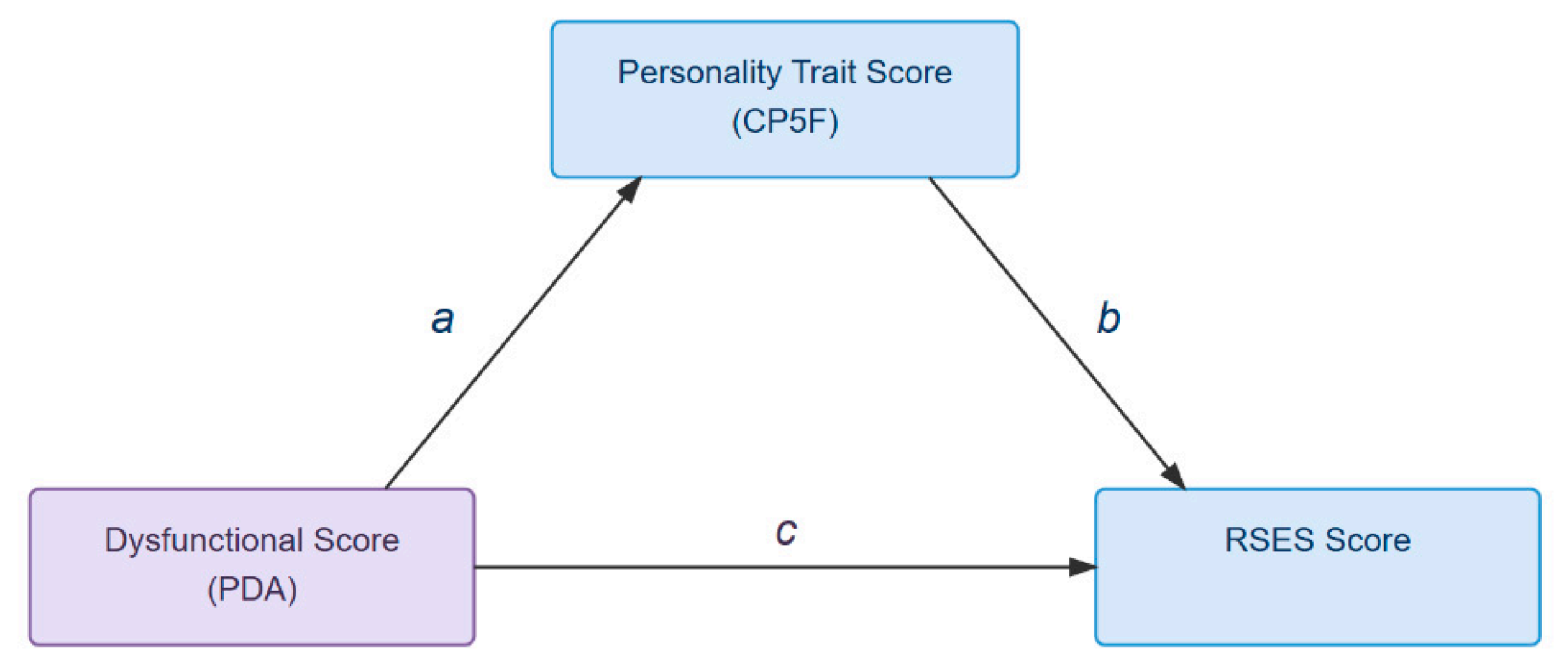
Conceptual mediation model. Note. a represents the path from the Dysfunctional Score (PDA) to the Personality Trait Score (CP5F); b represents the path from the Personality Trait Score (CP5F) to the RSES Score; c represents the total effect of the Dysfunctional Score (PDA) on the RSES Score, without controlling for the mediator.

For each mediation model (Hayes Model 4), linear regression assumptions regarding normality, linearity, homoscedasticity, and absence of multicollinearity were checked (Hayes, 2013). Their evaluation was carried out by inspecting the P–P plot graphs for normality, scatter plot of residuals and predicted values, as well as VIF (Variance Inflation Factor) indices, used for the evaluation of multicollinearity. The conceptual model is shown in Figure 2.

The direct effects were negative and significant in all five models, indicating that an increase in the Dysfunctional score was consistently associated with a decrease in the RSES score. Indirect effects were significant for Extraversion (ab = −0.026, β = −0.077, z = −4.064, 95% CI [−0.040, −0.015], *p* < 0.001), Conscientiousness (ab = −0.027, β = −0.079, z = −3.589, 95% CI [−0.044, −0.014], *p* < 0.001), Agreeableness (ab = −0.017, β = −0.051, z = −2.656, 95% CI [−0.032, −0.007], *p* = 0.008), and Emotional Stability (ab = −0.079, β = −0.232, z = −6.726, 95% CI [−0.104, −0.057], *p* < 0.001), with mediating effects ranging from low (Agreeableness) to medium to high (Emotional stability). Autonomy did not show a significant indirect effect (ab = −0.004, β = −0.013, 95% CI [−0.031, 0.010], *p* = 0.66), the confidence interval including zero; therefore, according to the criteria of Baron and Kenny (1986), mediation does not exist for this trait.

The detailed results are presented in Table 3.

### 3.5. The Parallel Mediation Model

**Figure 3:**
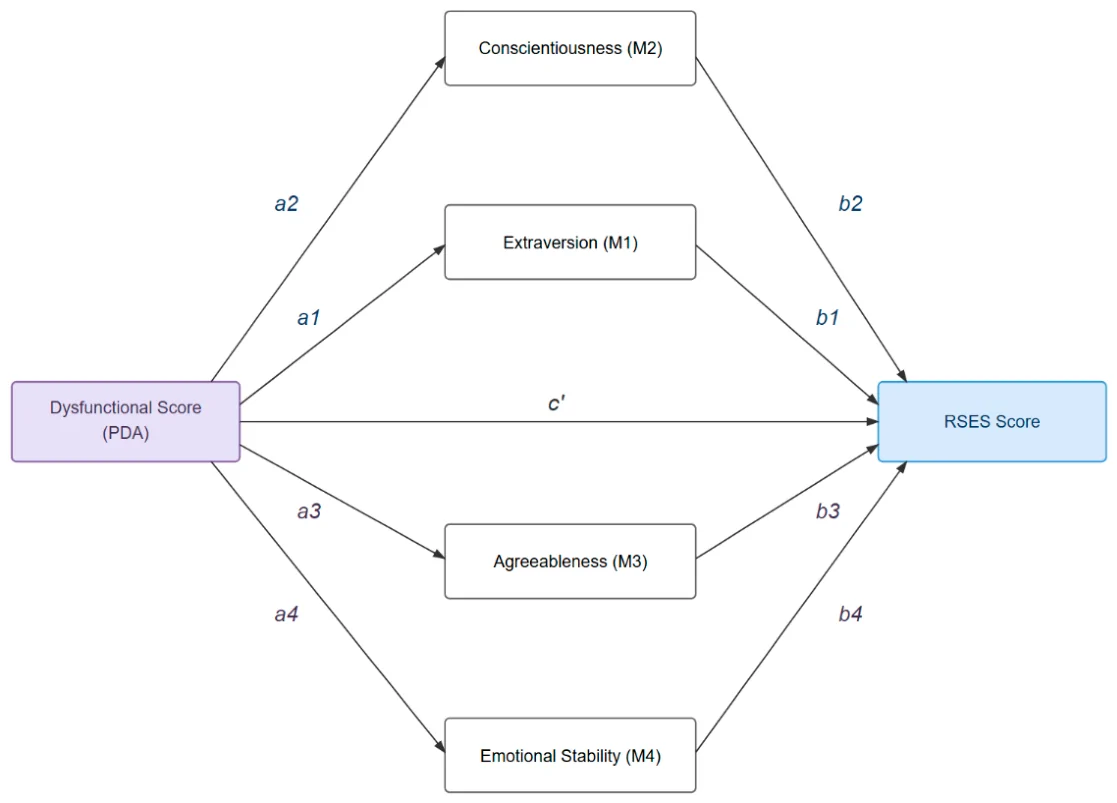
Parallel mediation—the conceptual model. Note. a1–a4 represent the paths from the Dysfunctional Score (PDA) to each mediator: a1 = path to Extraversion (M1); a2 = path to Conscientiousness (M2); a3 = path to Agreeableness (M3); a4 = path to Emotional Stability (M4). b1–b4 represent the paths from each mediator to the RSES Score: b1 = path from Extraversion (M1); b2 = path from Conscientiousness (M2); b3 = path from Agreeableness (M3); b4 = path from Emotional Stability (M4). c′ represents the direct effect of the Dysfunctional Score (PDA) on the RSES Score, after controlling for all mediators; c denotes the total effect when mediators are omitted.

Based on the previous results, a more complex mediation model was analyzed, in which all four relevant mediators were introduced into the model: Extraversion (M1), Conscientiousness (M2), Agreeableness (M3) and Emotional Stability (M4) as parallel mediators. The conceptual model is shown in Figure 3.

The analysis of the parallel mediation model highlighted that the Dysfunctional score (PDA) was negatively associated with Extraversion (a1), Conscientiousness (a2) and Emotional Stability (a4), with these relationships being statistically significant, while the association with Agreeableness (a3) was not significant.

Extraversion (b1), Conscientiousness (b2), and Emotional Stability (b4) showed positive and significant associations with RSES, while Agreeableness (b3) showed no significant association.

The results are presented in Table 4 and Table 5.

The indirect effects were negative and significant for Extraversion, Conscientiousness and Emotional Stability, while the indirect effect associated with Agreeableness was not significant. Both the direct effect (c′) and the total indirect effect were negative and significant, resulting in a significant total negative effect (Table 5).

Based on these results, the personality traits included in the model can be interpreted as parallel mediators of the relationship between the score of Dysfunctional negative emotions (PDA) and self-esteem (RSES), with the exception of Agreeableness, for which no significant mediating effect was observed.

To evaluate the robustness of the parallel mediation model to potential confounding, a supplementary analysis was conducted including age, marital status, and recidivism status (first-time conviction vs. repeat offender) as covariates. All specific indirect effects remained statistically significant, with the exception of the pathway through Agreeableness (consistent with the primary model), and the total indirect effect remained significant, supporting the robustness of the reported mediation pathways.

### 3.6. Positive Emotions (PDAs) and Personality Traits

As specified in H3, personality traits were also expected to relate to positive emotionality; the following analysis tests this component of the hypothesis. To examine the relationship between Positive Emotions and personality traits, a linear regression model was used in which the Positive Emotions score (PDA) was considered a dependent variable, and Extraversion, Conscientiousness and Agreeableness were included as predictors.

The results indicated positive and statistically significant associations for Extraversion and Conscientiousness, while Agreeableness was not a significant predictor of Positive Emotions. The model explained about 5% of the variability in Positive emotion scores (*R*^2^ ≈ 0.05), and the adequacy indicators suggested a satisfactory fit of the model. The regression coefficients, confidence intervals and significance levels are presented in Table 6. The performance of the model is shown in Table 7.

The model’s diagnosis indicated the absence of collinearity (VIF < 5) and autocorrelation (Durbin–Watson *d* = 1.77, is not substantially less than 2). The insignificant Breusch–Pagan test (*p* = 0.510) confirmed the hypothesis of homoscedasticity required in linear regression. The Shapiro–Wilk (*p* = 0.509) and Kolmogorov–Smirnov (*p* = 0.430) normality tests confirmed the normality hypothesis for residuals, a key requirement in linear regression.

**Figure 4:**
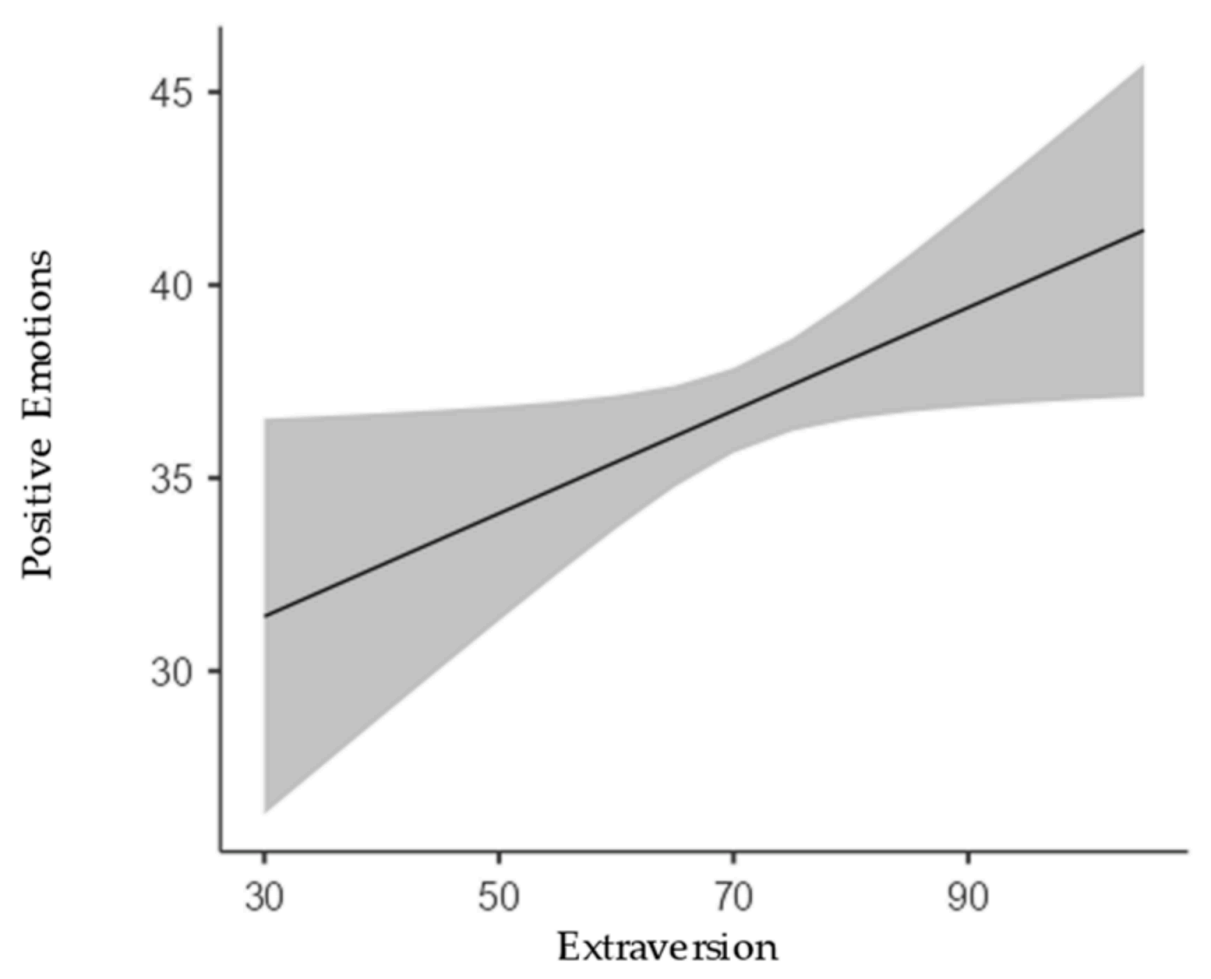
Estimating the marginal effect of Extraversion on Positive Emotions. Note. The solid line represents the estimated marginal effect. The shaded area represents the 95% confidence interval.

**Figure 5:**
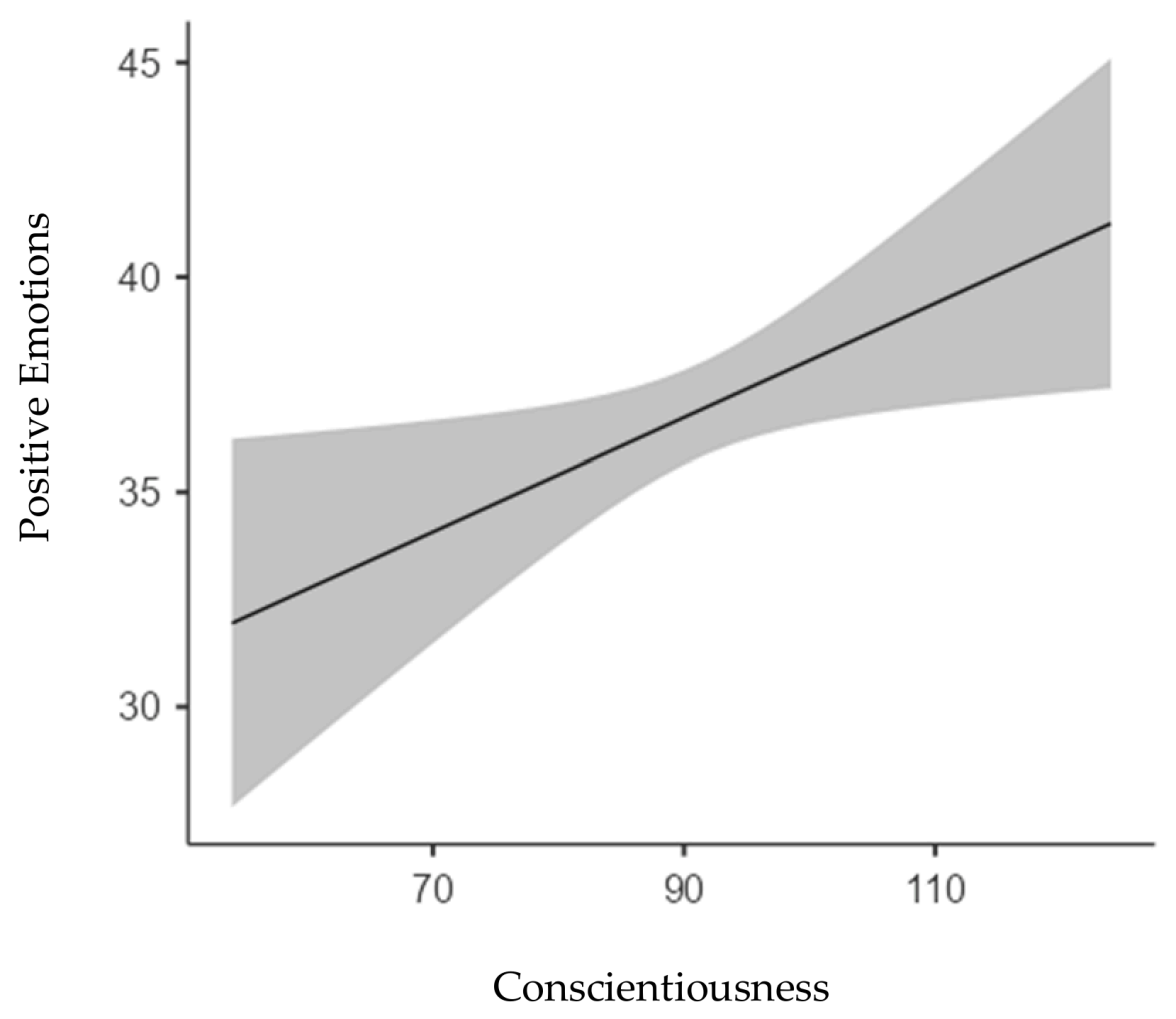
Estimating the marginal effect of Conscientiousness on Positive Emotions. Note. The solid line represents the estimated marginal effect. The shaded area represents the 95% confidence interval.

The model indicated that an increase in scores for Extraversion and Conscientiousness is associated with an increase in the score for Positive Emotions. Agreeableness was not significantly associated with Positive Emotions (Figure 4 and Figure 5).

### 3.7. Self-Esteem (RSES) and Personality Traits

To complement the mediation analysis and assess the discriminant contribution of personality traits beyond the mediators tested above, the direct relationship between self-esteem and the remaining CP5F dimensions was also examined. To examine the relationship between self-esteem and personality traits, a linear regression model was used in which the RSES score was considered a dependent variable, and Social Desirability, Extraversion, Emotional Stability and Autonomy were included as predictors. The model was statistically significant, *F*(4, 393) = 40.1, *p* < 0.001, explaining approximately 28% of the variability in self-esteem scores (*R*^2^ ≈ 0.28) (Table 8).

All predictors included in the model showed positive and statistically significant coefficients (*p* < 0.05), indicating that higher levels of personality traits are associated with higher self-esteem scores. Among these predictors, Emotional Stability had the strongest effect on the RSES score, with an increase of one unit associated with a 0.1349 unit increase in self-esteem.

The model’s diagnoses indicated the absence of multicollinearity problems (VIF < 5; Table 9) and autocorrelation (Durbin–Watson *d* = 1.95). In addition, the Goldfeld–Quandt (*p* = 0.990) and Harrison–McCabe (*p* = 0.987) tests supported the homoscedasticity hypothesis, and the Shapiro–Wilk (*p* = 0.216) and Kolmogorov–Smirnov (*p* = 0.958) normality tests indicated the normal distribution of residues.

To assess the discriminant validity of self-esteem relative to the CP5F traits, zero-order Pearson correlations were computed among self-esteem and the six personality dimensions (Table 10).

RSES correlated significantly with all six CP5F dimensions, most strongly with Emotional Stability (*r* = 0.488), Conscientiousness (*r* = 0.384) and Social Desirability (*r* = 0.377), and more weakly with Agreeableness (*r* = 0.294). While the correlation with Emotional Stability was the largest observed, it accounts for only about 24% of shared variance, indicating that self-esteem retains substantial unique variance not explained by any single personality trait, consistent with self-esteem functioning as a related but empirically separable construct rather than a redundant facet of the Big Five taxonomy (Judge et al., 1997; Erdle et al., 2009).

## 4. Discussion

### 4.1. Contextual Vulnerability: Placement History, Trauma, and Recidivism

Three indicators of psychosocial vulnerability converged on a similar interpretive pattern in the present data: a history of placement, traumatic experiences in the family of origin, and recidivism were each associated with elevated distress and/or reduced self-esteem. These findings are consistent with a shared developmental pathway, in which early relational instability—whether originating in placement, family trauma, or repeated negative labelling across the justice system—accumulates over time and may be reactivated under the conditions of confinement (Van Veldhuizen & Ingram, 2025; Blake et al., 2024; Moussavi et al., 2022; Engler et al., 2022; Turney & Wildeman, 2016; Krauss et al., 2020; Harris et al., 2015).

Specifically, participants with a history of placement reported significantly higher Depression and Total Distress scores, along with lower self-esteem, compared to those without this experience. Deprivation of liberty may be associated with the reactivation of these early vulnerabilities, potentially contributing to the more pronounced distress profile observed in this subgroup.

Young people who reported traumatic experiences in their family of origin (parental divorce, severe discipline, conviction of a parent) similarly showed higher levels of Sadness, Depression, and Total Distress (Zhang et al., 2021; Edwards Doh, 2023; Bendall et al., 2023), consistent with associations between early internalized self-blame and distress that may be amplified by the stigma and social adversity characteristic of the detention environment.

Recidivism followed a related pattern: participants at their first conviction achieved significantly higher self-esteem scores than repeat offenders. The literature on this association is mixed (Hubbard, 2006; Ghasimbaklo et al., 2014; Thapa et al., 2020; Boduszek et al., 2013); in the present sample, lower self-confidence among recidivists may be associated with the cumulative effect of negative labelling and compensatory reaffirmation of a criminal identity.

Taken together, these findings support the value of systematically assessing placement history, trauma exposure, and prior justice-system contact at intake, in order to identify young people at elevated risk of distress and impaired self-esteem early in the detention period.

### 4.2. Intimate Visits and Distress

Young people who benefited from intimate visits reported lower levels of Sadness and Total Distress, supporting the idea that physical and relational intimacy appears to function as a resource for emotional regulation even in conditions of deprivation of liberty (Berghuis et al., 2025; Mollaioli et al., 2021; Roy et al., 2021). Beyond the individual psychological dimension, reducing distress through intimate visits may have implications for the institutional climate: cited research suggests that access to such visits is associated with fewer incidents of sexual violence in detention (D’Alessio et al., 2013) and may contribute to reducing recidivism (Sanburn, 2014). The right to privacy, which goes beyond mere sexual activity, including basic needs for attachment and human contact, should be treated as a support for the mental health of prisoners, not as a discretionary privilege.

### 4.3. Clinical Severity: Chronic/Psychiatric Comorbidity and Suicide Risk

Two indicators of clinical severity—diagnosed chronic or psychiatric illness and a history of suicide attempts—were each associated with elevated distress across all PDA dimensions (Sadness, Depression, Concern, Anxiety, and Total Distress) and with lower self-esteem. This pattern is congruent with the literature identifying chronic illness and psychiatric comorbidity as robust predictors of psychological distress (Adzrago et al., 2024; Swathi et al., 2023; Xiao et al., 2022; Tareke et al., 2022) and negative self-assessment (Pinquart, 2013; Liu et al., 2019), as well as with the broader literature linking psychological distress to suicidal behavior (Wu et al., 2020; Owusu-Ansah et al., 2020; Rogers et al., 2025) and low self-esteem to suicide risk in young people (Soto-Sanz et al., 2019; Dat et al., 2021; Valladares-Garrido et al., 2026).

In the detention context, both indicators may be amplified by a common set of mechanisms: isolation, possible marginalization by fellow inmates, restricted access to treatment, and the identity strain associated with entry into the penitentiary system, particularly among first-time convicts. These conditions may be part of a pattern in which clinical vulnerability, social adversity, and distress are mutually reinforcing, and in which untreated symptoms could be associated with elevated risk of escalation toward serious incidents, including suicidal behavior.

Beyond the clinical dimension, affective distress symptoms (anxiety, depression) have also been linked to recidivism (Vasiljevic et al., 2017; Shishane et al., 2023). Taken together, these findings support the systematic screening of chronic/psychiatric diagnoses and suicide risk at intake, framing their proper clinical management not only as a humanitarian obligation but also as a component of both recidivism prevention (Wallace & Wang, 2020) and operational safety within detention facilities.

### 4.4. Concern, Depression and Self-Esteem

The regression model highlighted two relationships in opposite directions: Depression negatively predicted self-esteem, while Concern showed a surprising positive effect. The negative association between Depression and RSES is congruent with the bidirectional perspective in the literature, although longitudinal studies suggest that the effect of esteem on depression tends to be stronger than the reverse (Sowislo & Orth, 2013; Orth et al., 2009; Steiger et al., 2014; Gu et al., 2024; Kwok & Tam, 2023). In the context of detention, isolation and social rejection may co-occur with depression, which is, in turn, associated with a decline in self-image.

The positive effect of Concern on self-esteem differs from the negative associations reported in the general anxiety literature (Adiwijaya et al., 2024; Li et al., 2023; Maldonado et al., 2013), but it should be noted that Concern within PDA is a functional negative emotion, representing an adaptive form of apprehension. In the prison environment, concern about how you are perceived by colleagues, the staff of the unit or the parole board can motivate prosocial behaviors and can indirectly reinforce the feeling of personal worth. This hypothesis is worth exploring in future studies with longitudinal design.

### 4.5. Mediation of the Dysfunctional Distress Relationship—Self-Esteem Through Personality Traits

Mediation models indicated that dysfunctional distress is associated with lower self-esteem both directly and indirectly, with the indirect pathway operating through lower levels of Extraversion, Conscientiousness, and Emotional Stability. Autonomy did not function as a mediator in either model. Agreeableness showed a significant indirect effect in the individual mediation model, but this effect became non-significant in the parallel model. This pattern is consistent with shared variance among the personality mediators: when tested individually, Agreeableness may capture part of the variance attributable to the other traits, but its independent contribution appears limited once the remaining mediators are accounted for simultaneously.

A plausible explanation for the central mediating role of Extraversion, Conscientiousness, and Emotional Stability is that deprivation of liberty coincides with intensified dysfunctional distress, which is associated with a personality profile still in formation: young people scoring higher on dysfunctional distress tend to show lower emotional stability, lower organization, and greater social withdrawal, dimensions that are, in turn, related to self-esteem. Following a review of the available literature, no studies have been identified that have explicitly examined the mediating role of personality traits in the relationship between dysfunctional distress and self-esteem among young people deprived of liberty. From this perspective, the proposed model can be considered an original contribution to the understanding of the psychological mechanisms involved in socio-emotional adjustment in the prison context.

At the same time, the association of Extraversion and Conscientiousness with Positive Emotions suggests a reciprocal relationship between personality resources and positive affectivity, with direct implications for the design of rehabilitation interventions.

### 4.6. Self-Esteem and Personality Traits

Extraversion, Emotional Stability, Autonomy and Social Desirability showed significant positive associations with self-esteem, with Emotional Stability having the strongest effect. The result is consistent with the existing literature (Joshanloo, 2024; Al-Masri & Ma’abreh, 2020) and suggests that, in the context of detention, a more mature personality structure may be associated with less inconsistency in self-assessments. Taken together with the mediation results reported above, this suggests that, in the context of detention, greater personality maturation—and increased emotional stability in particular—may be associated with a more stable self-image and fewer destabilizing oscillations in self-assessment. However, given the modest-to-moderate size of the effects observed (e.g., β = −0.142 for the indirect pathway through Emotional Stability in the parallel model; *R*^2^ ≈ 0.28 for the direct regression model), these findings should be interpreted as indicating emotional stability as one relevant target among several, rather than as the primary or sufficient focus of psychological intervention. Strengthening emotional stability may usefully complement, rather than replace, interventions addressing self-esteem directly and the broader psychosocial vulnerabilities identified in this study.

### 4.7. Limitations of the Study

The study presents a series of methodological and interpretative limits that must be taken into account in the interpretation of the results. Firstly, the time window assessed by the PDA tool, limited to the last two weeks, restricts the possibility of generalising the results to the general state of the respondents, the responses may reflect the influence of particular recent events. Secondly, some divergent results from the existing literature, such as the direction of the association between Concern and self-esteem or the interpretation of the relationship between Depression and self-esteem, indicate the need for replication and expansion of the research. Third, the predominant use of non-parametric tests may limit the accuracy of estimates; however, the good or very good internal consistency of the instruments used, as well as the application of confirmatory factor analysis for some variables, are elements that support the accuracy of the results obtained. Fourth, data collection extended from November 2021 to 2024, a period overlapping with the final phase of COVID-19-related restrictions in Romania (state of emergency and subsequent state of alert, in effect until March 2022; Președintele României, 2020a, 2020b; Comitetul Naţional pentru Situaţii de Urgenţă, 2020, 2022), which suspended access to detention facilities and substantially delayed the onset of large-scale data collection. Consequently, the temporal distribution of the sample is uneven across years (*n* = 10 in 2021; *n* = 172 in 2022; *n* = 162 in 2023; *n* = 111 in 2024), reflecting logistical constraints rather than a planned longitudinal or cohort design. While this precludes formal testing of period effects, the pandemic-related disruption primarily affected the feasibility of access rather than the content of the instruments administered, and no systematic policy change specific to the study’s measures was identified during this interval; nonetheless, this temporal heterogeneity should be considered when interpreting the generalizability of the findings. Fifth, self-esteem was assessed using the RSES, which is subject to a well-documented ceiling effect at the upper end of the distribution; self-esteem was treated throughout as a continuous outcome, and no attempt was made to identify or interpret exceptionally high scores as reflecting narcissistic self-enhancement. Sixth, all constructs were assessed via self-report within a single administration, raising the possibility of common method bias (Podsakoff et al., 2003); this risk was partially mitigated by the use of distinct response formats and anchors across instruments (dichotomous items for trauma, a four-point Likert scale for the RSES, and five-point Likert scales for the PDA and CP5F), though it cannot be fully ruled out. Finally, the cross-sectional design precludes causal inference regarding the temporal ordering of distress, personality, and self-esteem; the mediation pathways reported should be interpreted as statistical associations consistent with, but not proof of, the proposed direction of effect.

## 5. Conclusions

The study investigated the relationships between affective distress, self-esteem, and major personality traits in a sample of 455 young adults deprived of liberty, while also examining the role of psychosocial vulnerability factors relevant to the rehabilitation process. The results indicate that placement history, diagnosis of chronic or psychiatric conditions, traumatic experiences, and suicide attempts are associated with higher levels of affective distress, and some of these variables also correlate with lower self-esteem. Access to intimate visits was linked to lower levels of distress, underscoring the role of close relationships in emotional regulation during incarceration.

With respect to the hypotheses formulated, the results partially confirm H1, H2, and H3. Psychosocial vulnerability factors were differentially associated with distress and self-esteem (H1). Dysfunctional components of distress negatively predicted self-worth, whereas Concern, as a functional emotion, showed an unexpected positive relationship with self-esteem (H2). Personality traits functioned both as direct predictors of self-esteem and positive emotionality and as mediators of the relationship between dysfunctional distress and self-esteem (H3).

Dysfunctional distress emerged as the central variable of the model: it was associated with lower self-esteem both directly and indirectly, through lower levels of Extraversion, Conscientiousness, and Emotional Stability, the latter showing the strongest mediating effect. Autonomy did not function as a mediator, and Agreeableness showed a non-significant effect in the parallel model. At the same time, Extraversion and Conscientiousness were positively associated with Positive Emotions, suggesting a reciprocal relationship between personality resources and positive affectivity, with direct implications for the design of rehabilitation interventions.

From a theoretical perspective, the study proposes an original parallel mediation model that explicitly tests the relationship between dysfunctional distress, personality traits, and self-esteem in the context of incarceration, contributing to the clarification of the mechanisms through which the penitentiary environment may affect the psychological functioning of young people during a sensitive period of identity formation. From a practical perspective, the results support the need for systematic initial screening upon entry into detention, targeting psychological, medical, and socio-familial vulnerabilities, followed by integrated interventions: psychotherapy focused on reducing dysfunctional distress, appropriate medical treatment in cases of comorbidity, and programs to strengthen emotional stability as a foundation for functional self-assessment.

## Figures and Tables

**Table 1:** Descriptive Statistics on Affective Distress (PDA).

Scale/Score	N	Min	Max	Mean	SD
Sadness	398	6	30	15.02	5.973
Depression	379	8	40	16.58	7.968
Concern	397	6	30	16.07	5.227
Anxiety	392	6	30	12.32	5.695
Positive	367	13	65	36.87	10.876
Functional	363	12	60	30.83	10.393
Dysfunctional	349	14	70	28.85	13.232
Total Distress	314	26	130	59.93	23.368

**Table 2:** Linear regression model.

Model		Non-Standard	Standard Error	Standardized	*t*	*p*	Tolerance	VIF
M_0_	(Intercept)	30.471	0.253		120.386	<0.001		
M_1_	(Intercept)	32.683	0.730		44.791	<0.001		
	Depression	−0.326	0.042	−0.574	−7.816	<0.001	0.465	2.149
	Concern	0.197	0.062	0.232	3.160	0.002	0.465	2.149

**Table 3:** Results of individual mediation models—significant mediators.

Mediator	a	βa	z	95% CI	*p*	b	βb	z	95% CI	*p*	ab	β (ab)	*R*^2^ Mediator	*R*^2^ RSES
Extraversion	−0.196	−0.283	−5.609	[−0.265, −0.127]	<0.001	0.134	0.272	5.183	[0.082, 0.188]	<0.001	−0.026	−0.077	0.080	0.223
Conscientiousness	−0.228	−0.215	−4.060	[−0.348, −0.120]	<0.001	0.117	0.365	7.683	[0.086, 0.147]	<0.001	−0.027	−0.079	0.156	0.283
Agreeableness	−0.198	−0.195	−3.356	[−0.319, −0.083]	<0.001	0.088	0.263	4.965	[0.052, 0.124]	<0.001	−0.017	−0.051	0.038	0.222
Emotional Stability	−0.395	−0.500	−10.158	[−0.469, −0.320]	<0.001	0.201	0.465	9.014	[0.157, 0.247]	<0.001	−0.079	−0.232	0.250	0.318

Note: a = effect of the predictor on the mediator; b = the effect of the mediator on the RSES; β = standardized coefficient; CI = confidence interval.

**Table 4:** Results of the parallel mediation model.

Mediator (M)	a	βa	z	95% CI	*p*	b	βb	z	95% CI	*p*
Extraversion (M1)	−0.195	−0.283	−5.244	[−0.269, −0.122]	<0.001	0.0934	0.189	3.902	[0.046, 0.140]	0.001
Conscientiousness (M2)	−0.228	−0.215	−3.920	[−0.343, −0.114]	0.001	0.0861	0.267	3.884	[0.042, 0.129]	0.001
Agreeableness (M3)	−0.198	−0.195	−3.542	[−0.324, −0.078]	<0.001	−0.0181	−0.054	−0.815	[−0.062, 0.026]	n.s.
Emotional Stability (M4)	−0.394	−0.499	−10.25	[−0.470, −0.319]	0.001	0.1230	0.285	4.60	[0.070, 0.176]	<0.001

Note: a = effect of the predictor on the mediator; b = the effect of the mediator on the RSES; β = standardized coefficient; CI = confidence interval; n.s. = non-significant.

**Table 5:** Indirect and global effects of the parallel mediation model.

Effect	Coefficient	β	z	IC 95% CI	*p*
a1b1 (Extraversion)	−0.0183	−0.054	−3.15	[−0.030; −0.008]	0.002
a2b2 (Conscientiousness)	−0.0197	−0.058	−2.78	[−0.035; −0.008]	0.006
a3b3 (Agreeableness)	0.0036	0.010	0.80	[−0.006; 0.014]	n.s.
a4b4 (Emotional Stability)	−0.0487	−0.143	−4.24	[−0.071; −0.028]	<0.001
Total indirect effect Σ(aibi)	−0.0831	−0.243	−6.49	[−0.108; −0.061]	<0.001
Direct Effect (c′)	−0.0516	−0.151	−2.94	[−0.092; −0.011]	0.003
Total Effect	−0.1347	−0.394	−7.66	[−0.170; −0.100]	<0.001

Note: a = effect of the predictor on the mediator; b = the effect of the mediator on the RSES; β = standardized coefficient; CI = confidence interval; n.s. = non-significant. Confidence intervals are percentile bootstrapped (1000 resamples). Standard errors, *z*-values and *p*-values are based on the delta method. Residual covariances among mediators were freely estimated.

**Table 6:** Coefficients of the linear regression model—positive emotions.

			95% CI					95% CI	
Predictor	Estimate	*SE*	Lower	Upper	*t*	*p*	Std. Estimate	Lower	Upper
Intercept	15.165	5.335	4.673	25.658	2.842	0.005			
Extraversion	0.133	0.062	0.011	0.256	2.144	0.033	0.111	0.009	0.213
Conscientiousness	0.132	0.056	0.021	0.245	2.336	0.020	0.179	0.028	0.330
Agreeableness	0.001	0.059	−0.114	0.118	0.030	0.976	0.002	−0.148	0.154

**Table 7:** Model performance.

							General Model Test			
Model	*R*	*R* ^2^	*R*^2^ Adjusted	AIC	BIC	RMSE	*F*	df1	df2	*p*
1	0.224	0.0502	0.0424	2783	2803	10.6	6.40	3	363	<0.001

**Table 8:** Linear regression model coefficients—RSES.

			95% Confidence Interval				
Predictor	Estimate	*SE*	Lower	Upper	*t*	*p*	*SE*
Intercept	7.4468	1.9916	3.5313	11.362	3.74	<0.001	
Social Desirability	0.0755	0.0247	0.0270	0.124	3.06	0.002	0.155
Extraversion	0.0571	0.0231	0.0116	0.102	2.47	0.014	0.116
Emotional Stability	0.1349	0.0233	0.0891	0.181	5.79	<0.001	0.315
Autonomy	0.0602	0.0205	0.0200	0.100	2.94	0.003	0.136

**Table 9:** Collinearity statistics.

	VIF	Tolerance
Social Desirability	1.41	0.708
Extraversion	1.22	0.821
Emotional Stability	1.64	0.610
Autonomy	1.18	0.848

**Table 10:** Zero-order correlations among self-esteem (RSES) and CP5F personality dimensions.

Variable	1	2	3	4	5	6	7
1. Extraversion	—						
2. Conscientiousness	0.220 ***	—					
3. Agreeableness	0.280 ***	0.750 ***	—				
4. Emotional Stability	0.480 ***	0.573 ***	0.565 ***	—			
5. Autonomy	0.351 ***	0.436 ***	0.273 ***	0.441 ***	—		
6. Social Desirability	0.273 ***	0.781 ***	0.773 ***	0.585 ***	0.400 ***	—	
7. RSES	0.300 ***	0.384 ***	0.294 ***	0.488 ***	0.317 ***	0.377 ***	—

*** *p* < 0.001. *N* = 393–399 (pairwise).

## Data Availability

The data presented in this study are available on request from the corresponding author. The data are not publicly available due to ethical restrictions and privacy concerns.

## Abbreviations

The following abbreviations are used in this manuscript:

CFA	Confirmatory Factor Analysis
CFI	Comparative Fit Index
CI	Confidence Interval
CP5F	5-Factor Personality Questionnaire
PDA	Affective Distress Profile
RMSEA	Root Mean Square Error of Approximation
RSES	Rosenberg Self-Esteem Scale
SE	Standard Error
TLI	Tucker–Lewis Index
VIF	Variance Inflation Factor
WLS	Weighted Least Squares
